# The Root Endophytic Fungi Community Structure of *Pennisetum sinese* from Four Representative Provinces in China

**DOI:** 10.3390/microorganisms7090332

**Published:** 2019-09-09

**Authors:** Zhen-Shan Deng, Xiao-Dong Liu, Bao-Cheng Zhang, Shuo Jiao, Xiang-Ying Qi, Zhi-Hong Sun, Xiao-Long He, Yu-Zhen Liu, Jing Li, Kai-Kai Chen, Zhan-Xi Lin, Ying-Ying Jiang

**Affiliations:** 1 College of Life Sciences, Yan’an University, Yan’an 716000, China (Z.-S.D.) (X.-D.L.) (X.-Y.Q.) (Z.-H.S.) (X.-L.H.) (Y.-Z.L.) (J.L.) (K.-K.C.); 2 School of Biological and Agricultural Science and Technology, Zunyi Normal College, Zunyi 53602, China; 3 State Key Laboratory of Crop Stress Biology in Arid Areas, College of Life Sciences, Northwest A&F University, Yangling 712100, Shaanxi, China; 4 National Engineering Research Center of Juncao, Fuzhou 350002, China

**Keywords:** *Pennisetum sinese*, endophytic fungi diversity, different ecoregion, forage grass

## Abstract

*Pennisetum sinese* is a good forage grass with high biomass production and crude proteins. However, little is known about the endophytic fungi diversity of *P. sinese*, which might play an important role in the plant’s growth and biomass production. Here, we used high throughput sequencing of the Internal Transcribed Spacer (ITS) sequences based on primers ITS5-1737 and ITS2-2043R to investigate the endophytic fungi diversity of *P. sinese* roots at the maturity stage, as collected from four provinces (Shaanxi province, SX; Fujian province, FJ; the Xinjiang Uyghur autonomous prefecture, XJ and Inner Mongolia, including sand (NS) and saline-alkali land (NY), China). The ITS sequences were processed using QIIME and R software. A total of 374,875 effective tags were obtained, and 708 operational taxonomic units (OTUs) were yielded with 97% identity in the five samples. Ascomycota and Basidiomycota were the two dominant phyla in the five samples, and the genera *Khuskia* and *Heydenia* were the most abundant in the FJ and XJ samples, respectively, while the most abundant tags in the other three samples could not be annotated at the genus level. In addition, our study revealed that the FJ sample possessed the highest OTU numbers (242) and the NS sample had the lowest (86). Moreover, only 22 OTUs were present in all samples simultaneously. The beta diversity analysis suggested a division of two endophytic fungi groups: the FJ sample from the south of China and the other four samples from north or northwest China. Correlation analysis between the environmental factors and endophytic fungi at the class level revealed that Sordariomycetes and Pucciniomycetes had extremely significant positive correlations with the total carbon, annual average precipitation, and annual average temperature, while Leotiomycetes showed an extremely significant negative correlation with quick acting potassium. The results revealed significant differences in the root endophytic fungi diversity of *P. sinese* in different provinces and might be useful for growth promotion and biomass production in the future.

## 1. Introduction

The Loess Plateau, which is located in the north of central China, is the largest loess accumulation plateau in the world and is one of the birthplaces of Chinese civilization. The Loess Plateau is one of the most severely eroded regions worldwide and has serious ecological problems. To solve these ecological problems, the Chinese government introduced the Grain to Green Program (GTGP), which aims to convert farmland to forest and grassland [1]. The implementation of GTGP has greatly improved the local ecological environment and increased the absorption and fixation of greenhouse gas carbon dioxide (CO2), which is of great significance to the environment of China and the world [2]. However, the implementation of GTGP has also caused some problems for social and economic development, such as a shortage of forage grass. *Pennisetum sinese*, a good bioenergy source, has been introduced to the Loess Plateau to increase the production of forage grass and has achieved remarkable results. The biomass production of *P. sinese* can reach up to 40 t ha^−1^ year^−1^ with a crude protein content of 15%–22% [3].

As an important forage grass, several studies have investigated the physiology and ecology of *P. sinese*, but little is known about the endophytic microbial community [3,4,5]. Endophytic microbes are considered to live inside a plant and do not cause host diseases [6,7]. Interactions between the endophytic microbes and the host plant can benefit both. The host can provide nutrients and shelter for endophytic microbes while the endophytic microbes can enhance the plant’s growth and stress resistance [7,8,9,10]. Thus, it is of great importance to investigate the endophytic microbes of *P. sinese* to promote its biomass production. Endophytic microbes include endophytic bacteria and endophytic fungi. Studies on endophytic bacteria of *P. sinese* from different provinces have shown great differences in different eco-regions of China, and soil properties and climate are the most important reasons behind these differences [11]. However, many studies on endophytes of *P. sinese* have evidenced a difference in the dominant bacteria [11,12].

Although some studies on the endophytic bacteria of *P. sinese* have been reported [11,12], there are still no reports on the root endophytic fungi community of *P. sinese.* Here, we ae the first to report the endophytic fungi diversity of *P. sinese* collected from four provinces of China (Shaanxi province, Fujian province, Xinjiang Uygur Autonomous Prefecture, and Inner Mongolia (including sandy and saline–alkali soil) using the high throughput sequencing method. The major objective of this study was to illuminate the endophytic fungi diversity and community of *P. sinese* from five representative provinces in China. 

## 2. Materials and Methods 

### 2.1. Sampling Sites and Sample Collection

**Figure 1:**
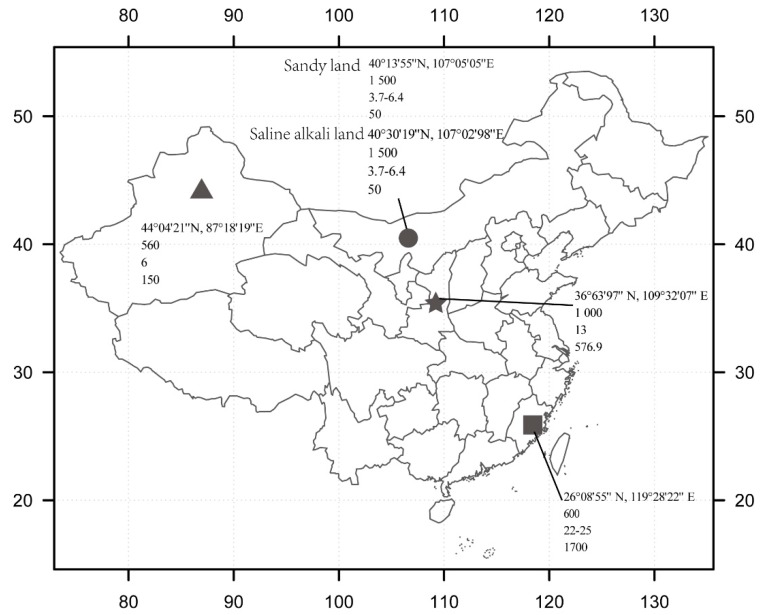
Geographical characteristics and climate types of the five sampling sites. Geographical characteristics of the five sampling sites are shown in the map (from the top to the bottom are latitude and longitude, altitude (m), annual average temperature (°C), and annual average precipitation (mm)). The triangle represents Changji, Xinjiang Uygur Autonomous Prefecture, which has a typical continental arid climate. The circle represents Bayannaoer, Inner Mongolia, which has a typical temperate continental climate. The pentagram represents Yan’an, Shannxi province, which has a typical warm temperate semi-humid and drought-prone climate. The square represents Fuzhou, Fujian province, which has a typical subtropical monsoon climate.

Root samples of *P. sinese* were collected during August to October 2018 at five sites in four eco-regions in China. The geographical characteristics of the sampling sites are shown in Figure 1, and the soil physicochemical properties are shown in Table 1. For sites in Bayannaoer, Inner Mongolia, sandy land and saline–alkali land were chosen for sample collection, because these two types of soil are both representative of their local environment. In each sampling site, five mature *P. sinese* plants were selected, and their healthy roots at different depths were cut down and stored in sterile tubes at 4 °C. For each plant root sample collection, a five-point sampling method was used. The root samples were then immediately transferred to the lab for further processing in less than 12 h. At the time of the root collection, soils around the roots were also collected to measure the soil’s physicochemical characteristics. To prevent the contamination of the soil and rhizosphere fungi, the root a surface sterilization procedure was performed, as previously described [11]. In order to verify the effect of surface sterilization, parts of the sterilized roots were rolled on potato dextrose agar (PDA) plates. Roots without fungi growth on the PDA plates were considered to be completely sterilized. Then, the root samples were stored at –80 °C until DNA extraction.

The physicochemical characteristics of the soil samples were measured using the protocols described by the USDA (1996) [13]. The altitudes and geographical coordinates were measured using a GPS locator (JIEWEISEN WS-009, Guangdong, China). The soil physicochemical properties are shown in Table 1.

### 2.2. DNA Extraction and Illumina Sequencing

The five root samples collected from the same site were mixed together and pooled as one sample. Approximately 300 g of the mixed root samples was used for DNA extraction. The genomic DNA was extracted using DNA quick plant system kit (Tiangen, Beijing, China), according to the manufacturer’s instruction, after gridding in liquid nitrogen. The genomic DNA’s concentration and purity were determined using Epoch (Bioteck, Winooski, VT, USA) and 1% agarose gel electrophoresis. The final concentration of each DNA sample was adjusted to 1.0 ng/µL using sterile distilled water. Primers ITS5-1737 (5′-GGAAGTAAAAGTCGTAACAAGG-3′) and ITS2-2043R (5′-GCTGCGTTCTTCATCGATGC-3′) were used to amplify the Internal Transcribed Spacer (ITS) sequences (about 300 bp). Novogene Co. Ltd. (Beijing, China) were entrusted to conduct the library construction and sequencing. Phusion* High-Fidelity PCR master mix with a GC buffer (New England Biolabs, Ipswich, UK) and an Eppendorf Gradient Thermocycler (Brinkman Instruments, Westbury, NY) were used for PCR amplification. PCR was carried out under the following conditions: initial denaturation at 95 °C for 180 s, 25 cycles of denaturation at 98 °C for 20 s, annealing at 55 °C for 15 s, extension at 72 °C for 15 s, and final extension at 72 °C for 1 min. The PCR amplicons were then tested using 2% agarose gel electrophoresis and pooled in equimolar ratios into a single tube. The target products were extracted using a Qiagen Gel Extraction Kit (Qiagen, Hilden, Germany), and, finally, the libraries were constructed using a TruSeq^®^DNA PCR-Free Sample Preparation Kit (Illumina, San Diego, CA, USA). The library was sequenced using an Illumina HiSeq 2500 platform and 2 × 250 bp paired-end reads were generated. 

### 2.3. Data Analysis

The raw reads first underwent quality control and length trimming procedures and were then divided into five groups according to their barcodes. The barcode sequences were truncated for further analysis. The remaining reads were then assembled to generate raw tags [14]; the clean tags were obtained by trimming the low quality raw tags [15,16]. The effective tags were generated using the clean tags after chimera sequence detection and removal [17].

Uparse (Uparse v7.0.1001, http://drive5.com/uparse/) [18] was used to cluster the effective tags and generate an operational taxonomic unit (OTU) with an identity threshold of 97%. The OTUs with only one sequence were removed, and the rest OTUs were annotated by the SSUrRNA database using Mothur [19]. 

The alpha diversity (including the observed species, the Shannon index; the Simpson index; the abundance-based coverage estimator (ACE); good-coverage; rarefaction analysis; rank abundance analysis) and beta diversity (including principal coordinate analysis (PCoA); and the unweighted pair-group method with arithmetic means (UPGMA)) were calculated by QIIME and displayed using the R software [16,20]. The correlation between environmental factors and endophytic fungi was calculated and plotted using R.

## 3. Results

### 3.1. Endophytic Fungal Community in Illumina Sequencing

A total of 385,438 raw tags were generated from the Illumina Miseq sequencing of the five samples. After quality control, a total of 375,609 clean tags remained. Then, after removal of the chimeras, 374,875 effective tags (ranging from 52,308 for XJ to 90,280 for NS) were obtained for OTU generation. The Q20 values for the five samples ranged from 99.18 to 99.38, indicating the high quality of the Illumina sequencing. An overview of the sequencing data is shown in Table 2. 

**Figure 2:**
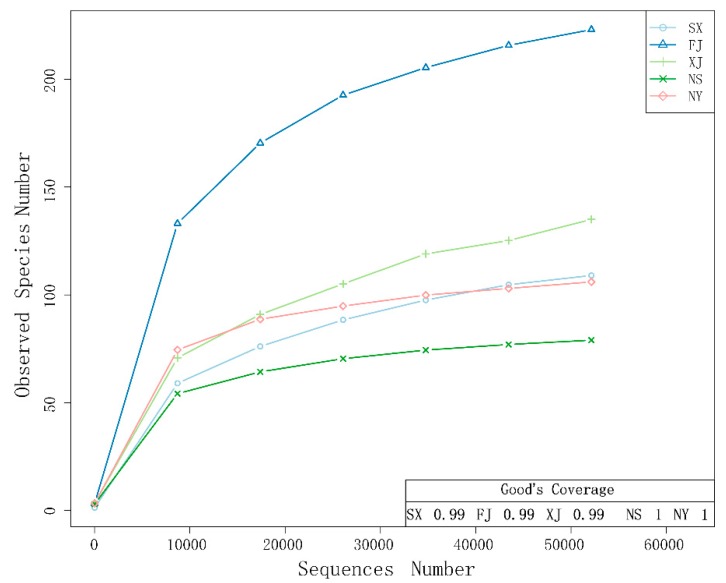
Rarefaction curves of the fungal community’s composition in five samples and Good’s coverage estimates (%). The rarefaction curves were assembled showing the number of operational taxonomic units (OTUs), clustered at a 97% sequence similarity cut-off in mothur, relative to the number of total sequences. The Good’s coverages of the five samples was calculated in mothur.

The flat trend of the latter part of the rarefaction curve indicates that the sequencing data were sufficient for each sample to represent the fungal communities (Figure 2). The rarefaction curves also showed the different abundance of fungal species. FJ had the highest abundance of fungal species, while NS had the lowest. The Good’s coverage values for the five samples ranged from 99% to 100% (Figure 2), indicating that the sequencing depth was great enough to capture most of the endophytic fungi in each sample.

**Figure 3:**
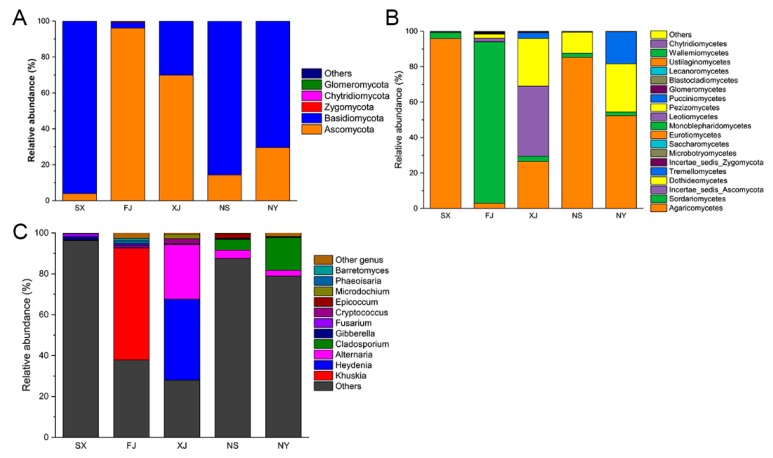
The relative abundance of endophytic fungi in the five samples at different taxonomy levels. (**A**): Relative abundance of fungi at the phylum level. (**B**): Relative abundance of fungi at the class level. (**C**): Relative abundance of fungi at the genus level with a relative abundance of more than 1%. Others in A, B, and C represent tags without annotations at specific level. Other genera in C represent tags that can annotate genera with a relative abundance of less than 1%.

Ascomycota and Basidiomycota were the predominant phyla in all samples. The relative abundance of Ascomycota and Basidiomycota could comprise up to 99% in each sample (Figure 3A). In SX, NS, and NY, the relative abundance of Basidiomycota was much higher than Ascomycota, while in FJ and XJ, the Ascomycota was higher than Basidiomycota. At the class level, Agaricomycetes were dominant in SX (95.9%), XJ (26.5%), NS (85.3%), and NY (52.4%), while the relative abundance of Agaricomycetes in FJ was only 2.9%. Sordariomycetes were dominant in FJ, with a relative abundance of 91.2%, while in other samples, they took up only 2% to 3.4%. Incertae_sedis_Ascomycota took up to 39.5% in XJ, while the relative abundance in other samples was lower than 2%. Dothideomycetes had a much higher relative abundance in XJ (26.9%), NS (11.9%), and NY (27.2%) than in SX (0.3%) and FJ (2.3%) (Figure 3B). In terms of genera, many tags that could not be annotated under known genera were named according to the others in Figure 3C. The relative abundance of the other genera in the five samples of SX, FJ, XJ, NS, and NY were 96.3%, 37.9%, 28.1%, 87.5%, and 78.9%, respectively. *Khuskia* was mainly found in FJ, which had a relative abundance of 54.7%. *Heydenia* was mainly found in XJ, with a relative abundance of 39.5%. *Alternaria* had a higher relative abundance in XJ (26.7%) and a relative lower abundance in NS (4.1%) and NY (2.8%). *Cladosporium* was mainly found in NS and NY, with a relative abundance of 5.3% and 16.0%, respectively. Other genera with a relative abundance lower than 10% in any of the five samples are not presented specifically here.

**Figure 4:**
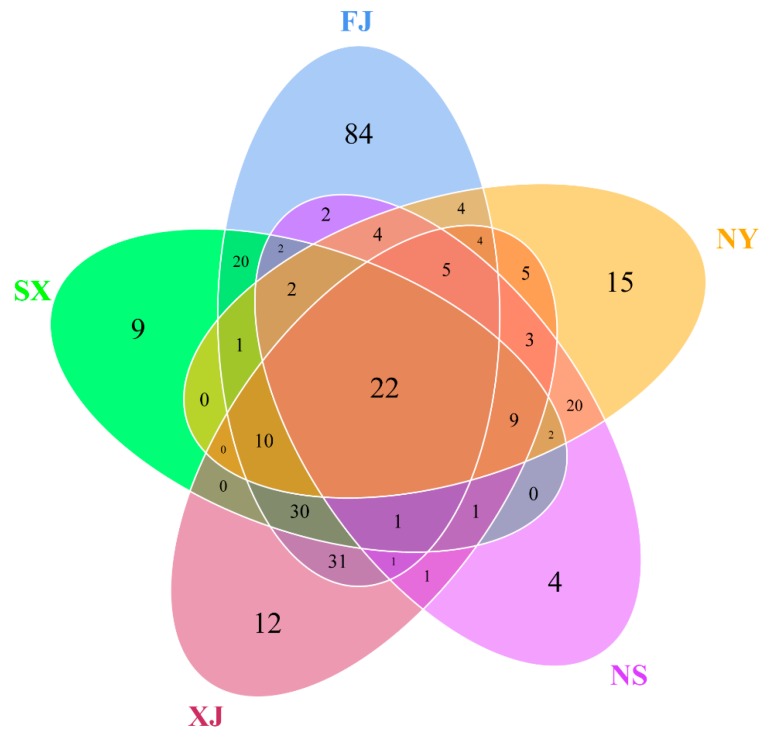
Venn diagram of the five *P. sinese* samples. The numbers inside the diagram indicate the number of OTUs. A total of 9, 84, 12, 4, and 15 unique OTUs were found in SX, FJ, XJ, NS, and NY, respectively, and 22 OTUs were shared by all five samples.

A total of 708 OTUs were obtained from the high-throughput sequencing, with 130, 242, 135, 86, and 115 for SX, FJ, XJ, NS, and NY, respectively. The OTU distribution in the five sample is shown in the Venn diagram (Figure 4). A total of 22 OTUs were shared by all five samples, and the unique OTUs for SX, FJ, XJ, NS, and NY were 9, 84, 12, 4, and 15, respectively.

### 3.2. Alpha Diversity Analysis

The alpha diversity indices of the five samples are listed in Table 3. A whole tree of the highest observed species, ACE and PD Whole Tree, occurred in the FJ sample, and the index values were 223, 246.86, and 57.80. In the contrast, a whole tree of the lowest observed species, ACE and PD, occurred in the NS sample, and the values were 79, 89.13, and 15.89. The NY sample had the highest Shannon index (2.43), while the lowest index was found in the SX sample at 0.56. The highest Simpson index occurred in the XJ sample at 0.71 while the lowest Simpson index was found in the SX sample at 0.12. The XJ sample possessed the highest Chao1 index at 238.50, while the NS sample possessed the lowest Chao1 index at 83.58.

### 3.3. Beta Diversity Analysis

**Figure 5:**
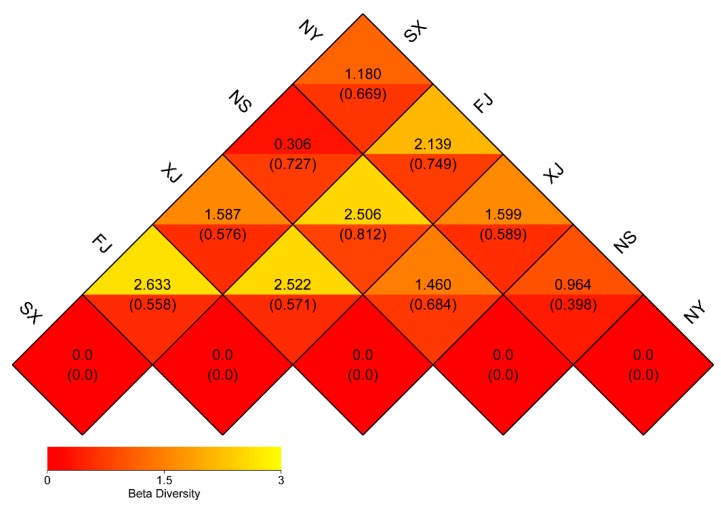
Heatmap of the dissimilarity coefficient between the five *P. sinese* samples. In the same square, the upper and lower values represent the coefficients calculated based on the Weighted Unifrac matrix and Unweighted Unifrac distance matrix.

The dissimilarity coefficient between the five samples was calculated based the Weighted Unifrac and Unweighted Unifrac distance matrix (Figure 5). The smaller the dissimilarity coefficient, the smaller the difference between the two samples. Based on the Weighted Unifrac distance matrix, the FJ sample had the highest dissimilarity with the SX sample at 2.663, while the NS sample had the lowest dissimilarity with the SX sample at 0.306. However, there was little difference in the dissimilarity coefficient calculated based on the Unweighted Unifrac distance. The dissimilarity coefficient between the FJ and NS samples was the highest at 0.812 and was the lowest, at 0.398, between NY and NS. 

**Figure 6:**
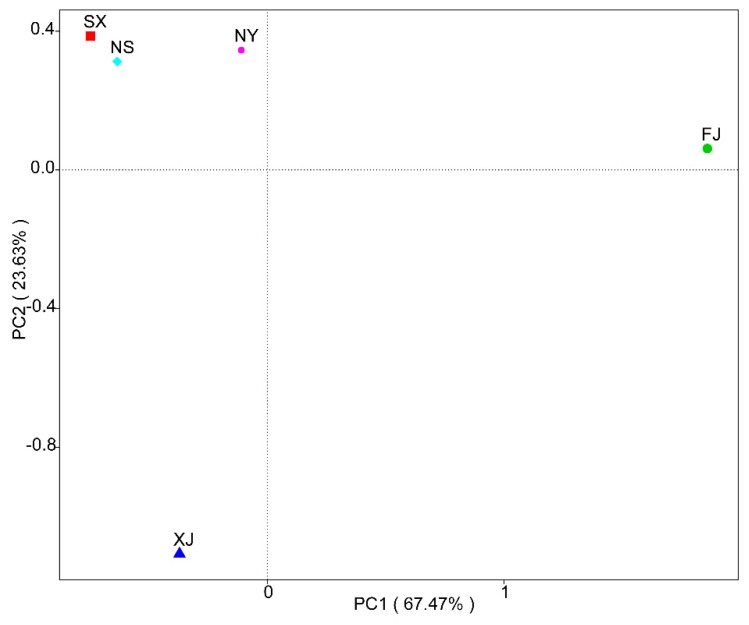
Principal coordinate analysis (PCoA) plot based on the weighted Unifrac distance matrix for the five *P. sinese* samples.

**Figure 7:**
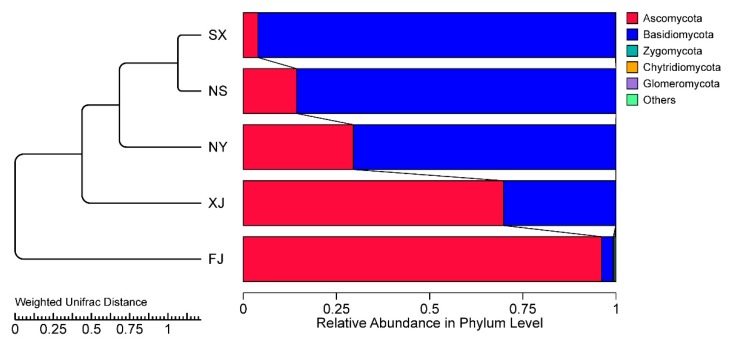
Unweighted Pair-group Method with Arithmetic Mean (UPGMA) clustering tree based on the weighted Unifrac distance matrix. The relative abundances of the top ten phyla in all samples are indicated, and the rest of the phyla are indicated as “Others”.

**Figure 8:**
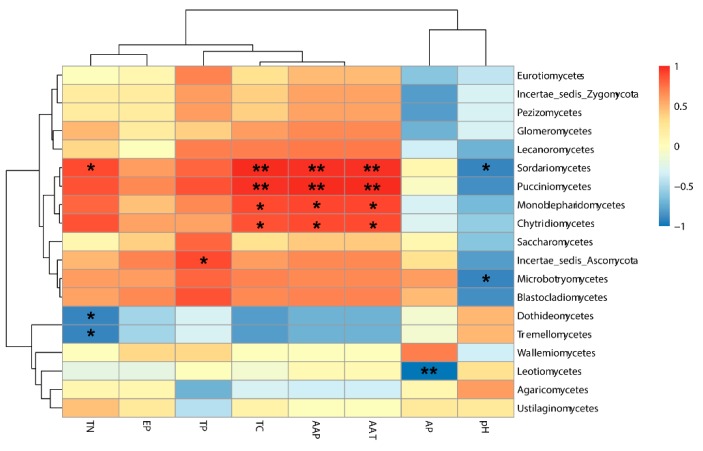
Heatmap of correlation between the environmental factors and the endophytic fungi at the class level. * and ** represent the *p* < 0.05 and *p* < 0.01, respectively. TN, EP, TP, TC, AAP, AAT, and AP represent total nitrogen, effective phosphorus, total phosphorus, total carbon, annual average precipitation, annual average temperature, and quick acting potassium, respectively.

A principal coordinate analysis (PCoA) based on the weighted Unifrac distance matrix was conducted to show the relationship between the different *P. sinese* samples (Figure 6). In the PCoA result, the first axis explained 67.47% of the data’s variability and the second axis explained 23.63%. The distance between the NS and SX was the smallest, and the FJ sample possessed the largest distance from other samples. The Unweighted Pair-group Method with Arithmetic Mean (UPGMA) method was used to cluster the five samples based on the weighted Unifrac distance matrix (Figure 7). The result revealed that SX and NS were clustered together while FJ had the largest difference compared to other samples in its fungi diversity, which is similar to the results of the PCoA. Moreover, a correlation between the environmental factors and the endophytic fungi at the class level was conducted (Figure 8). Sordariomycetes and Pucciniomycetes possessed an extremely significant positive correlation with the total carbon (TN), annual average precipitation (AAP), and annual average temperature (AAT) while Monoblepharidomycetes and Chytridiomycetes possessed a significantly positive correlation with TN, AAP, and AAT. Sordariomycetes and Incertae_sedis_Zygomycota possessed a significantly positive correlation with the total nitrogen and total phosphorus, respectively. Leotiomycetes possessed an extremely significant negative correlation with quick acting potassium (AP). Sordariomycetes and Microbotryomycetes possessed a significantly negative correlation with pH while Dothideomycetes and Tremellomycetes possessed a significantly negative correlation with TN. 

## 4. Discussion

Because *P. sinese* is an important feedstock in graziery and agroforestry in many places in China, it is important to study its endophytic microbial community. Many reports have suggested that endophytic fungi could promote host growth, tolerance to biotic and abiotic factors, and nutrient absorption [21,22,23,24]. Our study focused on the root endophytic fungi diversity of *P. sinese* collected from five provinces in China. Investigation of the endophytic fungi of *P. sinese* roots does not only provide information needed for further study but also provides an understanding of the differences in the endophytic fungal diversity in different environments. Meanwhile, a better understanding of root endophytic fungi could help us better understand the interactions between the fungi and *P. sinese* and promote the production and quality of forage grass.

To investigate the root endophytic fungi diversity of *P. sinese*, a high throughput method was used identify the ITS1 sequence of *P. sinese* root samples, and a total of 798 OTUs, ranging from 79 in the NS sample to 223 in the FJ sample, were identified (Table 1). Ascomycota and Basidiomycota were the two dominant phyla in the five samples. This phenomenon has also been shown by other researchers [25,26,27,28]. In the root of *P. sinese* collected from Yanan, Shannxi province, most of the sequencing tags (94%) belonged to phylum Basidiomycota and order Russulales, but the tags could not be annotated to the family level. The same situation also occurred for the root samples collected from Bayannaoer, Inner Mongolia, in both sandy land and saline–alkali land. The dominant unknown fungi sequenced SX, NS, and NY samples indicate the need for further study of root endophytic fungi. The high abundance of unknown fungi may play an important role in the growth of *P. sinese* in Shannxi province and Inner Mongolia. The most dominant OTU in the FJ sample belonged to species *Khuskia oryzae*. Some species of genus *Khuskia* are plant pathogens [29]. However, studies on *Khuskia oryzae* are still rare. The high abundance of *K. oryzae* in the root of *P. sinese* collected from Fuzhou, Fujian province, indicates the important, yet unknown, roles that *K. oryzae* play in the growth of *P. sinese*. 

The results of the alpha diversity analysis revealed a higher diversity of endophytic fungi in the FJ sample than in the other four samples (Table 3). In addition, the PCoA analysis showed an obvious division, in the first axis, between sample group of SX, XJ, NS and the FJ sample (Figure 6). The higher rainfall and temperature might be the main cause of this difference. Meanwhile, the correlation analysis (using the Spearman method) between the endophytic fungi at the class level and environmental factors also revealed an extremely significant positive relationship between the AAP and AAT and Sordariomycetes and Pucciniomycetes, which occupied a much higher proportion in the FJ sample than in the four other samples (Figure 8). The UPGMA analysis also suggested that the FJ sample is significantly different from the other four samples. Our results also revealed that the endophytic fungi diversity in the south of China (the FJ sample) was different from the diversity in north and northwest China (the NS, NY, SX, and XJ samples). Previous studies have already demonstrated that both the climatic conditions and soil physicochemical properties might affect the diversity and composition of the endophytic microbes [30,31,32]. The correlation analysis results also revealed that both the climatic conditions and soil physicochemical properties had significant influence on certain fungi at the class level (Figure 8). Although the climate was the same for the NS and NY samples, there were still obvious differences in their endophytic fungi diversity (Figure 3B and Figure 8). In addition, the endophytic fungi composition of the NS sample showed a closer relationship to the SX sample than to the NY sample (Figure 6), which suggests the influence of soil’s physicochemical properties on fungi diversity. Our results, then, are consistent with the previous reports and demonstrate that both soil characteristics and climate factors play an important role in determining endophytic fungi diversity and community composition.

In conclusion, this article is the first to report the diversity of endophytic fungi of *P. sinese* from four ecoregions in China. Alpha diversity and beta diversity analyses revealed that the endophytic fungi structure and composition was different in the roots of *P. sinese* collected from different ecoregions, and the soil’s physicochemical properties and climate factors had important impacts on endophytic fungi diversity and the abundance of *P. sinese* in the different samples. Isolation and function studies of these endophytic fungi are required to understand the role the fungi play in plant growth. This study provides the basic information we need to understand the endophytic fungi diversity of *P. sinese* and might help improve the growth, production, and quality of *P. sinese*.

## Figures and Tables

**Table 1:** The physicochemical characteristics of the five soil samples.

Sampling Sites	SX	FJ	XJ	NS	NY
	Yanan, Shaanxi Province	Fuzhou, Fujian Province	Changji, Xinjiang Uygur Autonomous Prefecture	Bayannaoer, Inner Mongolia (Sandy Land)	Bayannaoer, Inner Mongolia (Saline Alkali Land)
Total carbon (%)	2.47	4	1.44	0.77	0.23
Total nitrogen (%)	0.62	0.53	0.24	0.12	0.04
Total phosphorus (mg/kg)	1784	2065	2036	1465	1718
Effective phosphorus (mg/kg)	12.6	10	10.8	3	3.5
Quick acting potassium (mg/kg)	132.9	112.8	324.7	154.1	99.5
pH	8.35	6.98	8.27	8.9	9.38

**Table 2:** Overview of the sequencing data.

Sample Name	Raw Tags	Clean Tags	Effective Tags	Base (nt)	Average Length (nt)	Q20	GC%	Effective%
SX	79,608	77,989	77,902	20,915,412	268	99.27	56.08	97.02
FJ	79,018	78,476	78,175	16,975,477	217	99.38	49.92	97.33
XJ	52,987	52,439	52,308	12,849,443	246	99.18	45.34	97.65
NS	94,083	90,362	90,280	23,771,714	263	99.34	55.38	95.27
NY	79,742	76,343	76,210	18,856,088	247	99.36	51.65	94.37

**Table 3:** Alpha diversity indices of the five samples.

Sample Name	Observed_Species	Shannon	Simpson	Chao1	ACE	PD_Whole_Tree
SX	109	0.56	0.12	130.37	136.71	29.92
FJ	223	2.12	0.60	237.88	246.86	57.80
XJ	135	2.205	0.71	238.50	179.16	32.84
NS	79	1.19	0.30	83.58	89.13	15.89
NY	106	2.43	0.69	110.13	113.46	21.33
